# Mitochondrial dysfunction following repeated administration of alprazolam causes attenuation of hippocampus-dependent memory consolidation in mice

**DOI:** 10.18632/aging.205087

**Published:** 2023-10-05

**Authors:** Siqing Zhu, Jingjing Shi, Qian Jin, Yi Zhang, Ruihua Zhang, Xuejun Chen, Chen Wang, Tong Shi, Liqin Li

**Affiliations:** 1 State Key Laboratory of NBC Protection for Civilian, Changping, Beijing 102205, China

**Keywords:** proteomics, alprazolam, repeated administration, mitochondrial damage, memory consolidation

## Abstract

The frequently repeated administration of alprazolam (Alp), a highly effective benzodiazepine sedative-hypnotic agent, in anxiety, insomnia, and other diseases is closely related to many negative adverse reactions that are mainly manifested as memory impairment. However, the exact molecular mechanisms underlying these events are poorly understood. Therefore, we conducted a proteomic analysis on the hippocampus in mice that received repeated administration of Alp for 24 days. A total of 439 significantly differentially expressed proteins (DEPs) were identified in mice with repeated administration of Alp compared to the control group, and the GO and KEGG analysis revealed the enrichment of terms related to mitochondrial function, cycle, mitophagy and cognition. *In vitro* experiments have shown that Alp may affect the cell cycle, reduce the mitochondrial membrane potential (MMP) to induce apoptosis in HT22 cells, and affect the progress of mitochondrial energy metabolism and morphology in the hippocampal neurons. Furthermore, *in vivo* behavioral experiments including IntelliCage System (ICS) and nover object recognition (NOR), hippocampal neuronal pathological changes with HE staining, and the expression levels of brain-deprived neuron factor (BDNF) with immunohistochemistry showed a significant decrease in memory consolidation in mice with repeated administration of Alp, which could be rescued by the co-administration of the mitochondrial protector NSI-189. To the best of our knowledge, this is the first study to identify a link between repeated administration of Alp and mitochondrial dysfunction and that mitochondrial impairment directly causes the attenuation of memory consolidation in mice.

## INTRODUCTION

Insomnia is a widespread sleep disorder, often mutually reinforced by anxiety [1]. Alprazolam (Alp) is a classic benzodiazepine (BDZ) that remains the first-line clinical insomnia treatment [2], especially for patients with anxiety-like insomnia [3]. The most common adverse effects of Alp include drowsiness, dizziness, and memory loss [4]. Data from the Food and Drug Administration Adverse Event Reporting System extracted BDZs-related dementia events, of which Alp accounted for 17% of these events [5].

Although several studies have shown that Alp causes cognitive dysfunction, such as memory loss [6], the specific mechanisms remain elusive. Memory loss can occur at different stages of memory learning, including acquisition, consolidation, reconsolidation, and other processes [7–9]. Moreover, the most typical memory loss is hippocampus-dependent spatial learning memory disorder. Obviously, memory loss is also the main clinical symptom of many neurodegenerative diseases such as Alzheimer’s disease (AD) [10]. And, the hippocampus, a brain area critical for learning and memory, is especially vulnerable to damage at early stages of AD [11]. Recently, through high-throughput techniques such as transcriptomics, metabolomics and proteomics, many studies have attempted to discover differential hallmarks in the hippocampus of AD, including dysregulation of stress signaling [12] and synaptic signaling [13], disorders of serine metabolism [14] and mitochondrial dysfunction [15].

As the primary energy producer, the quality of mitochondria is crucial to the survival and proper function of neurons [16]. Furthermore, many studies have indicated that abnormal mitochondrial bioenergetics is a major pathological feature of several neurodegenerative diseases including AD and Parkinson’s [17–19]. Mitochondrial dysfunction is not only related to abnormal energy generation of neurons [20], but also to abnormal reactive oxygen species (ROS) homeostasis [21], Ca^2+^ imbalance [22], apoptosis abnormalities [23], dynamical morphology [24], mitochondrial DNA changes [25], and autophagy [26]. Accumulating evidence indicates that mitochondrial damage is closely linked to memory loss [27]. Therefore, we hypothesized that mitochondrial dysfunction might be associated with Alp-induced memory loss.

Therefore, to further validate our hypothesis, we first performed proteomic analysis on the hippocampus, subsequently, the detrimental effects of Alp on apoptosis, cycle, MMP, energy metabolism and morphology were verified, and finally, the fact that Alp’s effect of attenuating memory consolidation could be resisted by mitochondrial protectors was demonstrated. To the best of our knowledge, this is the first study to disclose direct evidence between repeated administration of Alp and mitochondrial dysfunction, which is a putative factor in cognitive impairment caused by repeated Alp administration. The current study proposed a novel mechanism by which Alp causes cognitive impairment, which might provide new insights in the clinical safety of Alp.

## RESULTS

### Repeated administration of Alp causes differential expression of mitochondrial functional proteins

Since the hippocampus is inextricably linked to memory acquisition, consolidation, and extraction, a proteomic method was utilized to identify protein changes that occur in the hippocampus of mice with repeated administration of Alp.

**Figure 1:**
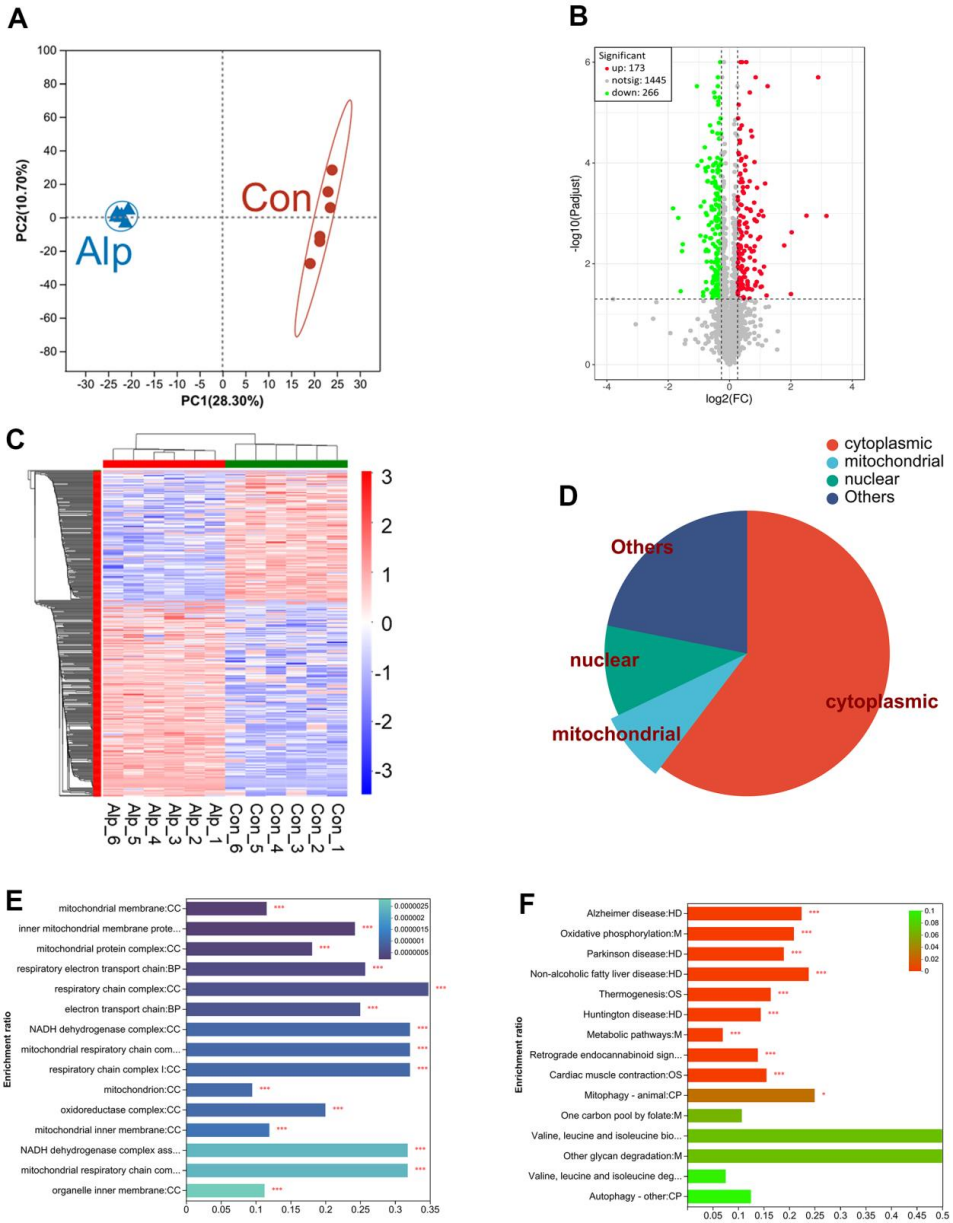
**Proteomic analysis of the hippocampus of mice following repeated administration of Alp (n = 6 per group).** (**A**) The PCA analysis of the protein profiles. (**B**) Volcano map of the expression of all DEPs, which red indicating upregulation and green indicating downregulation. (**C**) Heat map demonstrating DEPs expression levels of Alp-treated animals versus controls, with red indicating upregulation and blue indicating downregulation. (**D**) The proportion of distribution of DEP in subcellular locations such as cytoplasm, nucleus, and mitochondrial. (**E**) GO analysis demonstrates the biological processes and protein functions in which mitochondrial subcellular localization proteins were involved. (**F**) The KEGG results of DEPs were identical to 15 pathways in the KEGG results of network pharmacology. The different colors of the columns represent p-value.

The principal component analysis (PCA) can adequately distinguish between the control and Alp groups (Figure 1A). In the DIA proteomics study, > 1869 proteins were identified and quantified with acceptable confidence. Among these, the expression of 439 proteins changed significantly, including 173 upregulated and 266 downregulated proteins (Figure 1B). The information for these 439 DEPs is shown in Supplementary Table 1.

As shown in Figure 1C, cluster heat map was generated for the 439 DEPs with significant changes in relative abundance. The distinction between the control and Alp groups showed that repeated Alp exposure significantly affected the biological processes in the hippocampus.

Among these, we have focused on subcellular localization of DEPs mainly in the cytoplasm, nucleus, and mitochondria (Figure 1D). Moreover, combined with the DEPs Supplementary Table 1, 7 upregulated mitochondrial proteins were identified: cytochrome c oxidase subunit IV (COX 4), cytochrome c oxidase subunit VIa (COX 6A), complex III subunit 5 (RISP), NADH dehydrogenase subunit 5 (ND5), acyl carrier protein (Ndufab1), NADH dehydrogenase [ubiquinone] 1 alpha subcomplex subunit 13 (Ndufa13), and optic atrophy protein 1 (OPA1). In addition, 14 downregulated mitochondrial proteins were identified, including cytochrome c oxidase subunit 6B (COX 6B), cytochrome c oxidase subunit 6C (COX 6C), succinate dehydrogenase complex subunit (CybS), fumarase, complex III subunit 3 (Cob), ATP synthase protein 8 (ATP8), mitochondrial pyruvate carrier 2 (MPC2), NADH dehydrogenase subunit 4 (ND4), NADH dehydrogenase [ubiquinone] iron-sulfur protein 6 (Ndufs6), NADH dehydrogenase [ubiquinone] iron-sulfur protein 5 (Ndufs5), NADH dehydrogenase [ubiquinone] 1 subunit C2 (Ndufc2), NADH dehydrogenase [ubiquinone] 1 beta subcomplex subunit 7 (Ndufb7), NADH dehydrogenase [ubiquinone] 1 beta subcomplex subunit 10 (Ndufb10), and mitochondrial fission 1 protein (FIS1) (Table 1).

**Table 1:** KEGG terms enriched from significantly regulated mitochondria-associated proteins between mice with Alp and control groups.

**Accession no**	**Protein**	**Mw (kDa)**	**FC**	**p-value**
P19783	COX 4	19.5	1.289	0.00080
P43024	COX 6A	12.5	1.862	0.00019
P56391	COX 6B	10.1	0.814	0.00013
P56391	COX 6C	8.5	0.799	0.00024
Q9CXV1	CybS	17	0.755	0.00007
P97807	Fumarase	54.3	0.773	0.00007
Q9CR68	RISP	29.3	1.239	0.00271
P00158	Cob	43.2	0.816	0.00712
P03930	ATP8	7.8	0.817	0.00056
Q9D023	MPC2	14.3	0.820	0.00060
P03911	ND4	51.8	0.124	0.00904
P03921	ND5	68.4	1.243	0.02997
P52503	Ndufs6	13	0.732	0.02905
Q99LY9	Ndufs5	12.6	0.670	0.00241
Q9CQ54	Ndufc2	14.2	0.562	0.00210
Q9CR21	Ndufab1	17.4	1.579	0.00024
Q9CR61	Ndufb7	16.3	0.806	0.01188
Q9DCS9	Ndufb10	21	0.825	0.02277
Q9ERS2	Ndufa13	16.8	1.281	0.00026
Q9CQ92	FIS1	17	0.755	0.00508
P58281	OPA1	115.5	1.335	0.00033

Further, GO enrichment analysis (Figure 1E) displayed significant changes in subcellular localization of DEPs in mitochondria. It mainly affects cellular components including mitochondrial membrane, mitochondrial protein complex, respiratory electron transport chain, NADH dehydrogenase complex and oxidoreductase complex.

The KEGG pathway analysis was shown in Figure 1F. Totally, 15 of the main pathways were presented, and the top one was metabolic pathways, such as oxidative phosphorylation and other metabolic process including folate, glycan and degradation of valine, leucine, and isoleucine. In addition, many DEPs were enriched in the neurodegenerative diseases’ pathway, including Alzheimer’s, Parkinson’s, and Huntington’s diseases.

**Figure 2:**
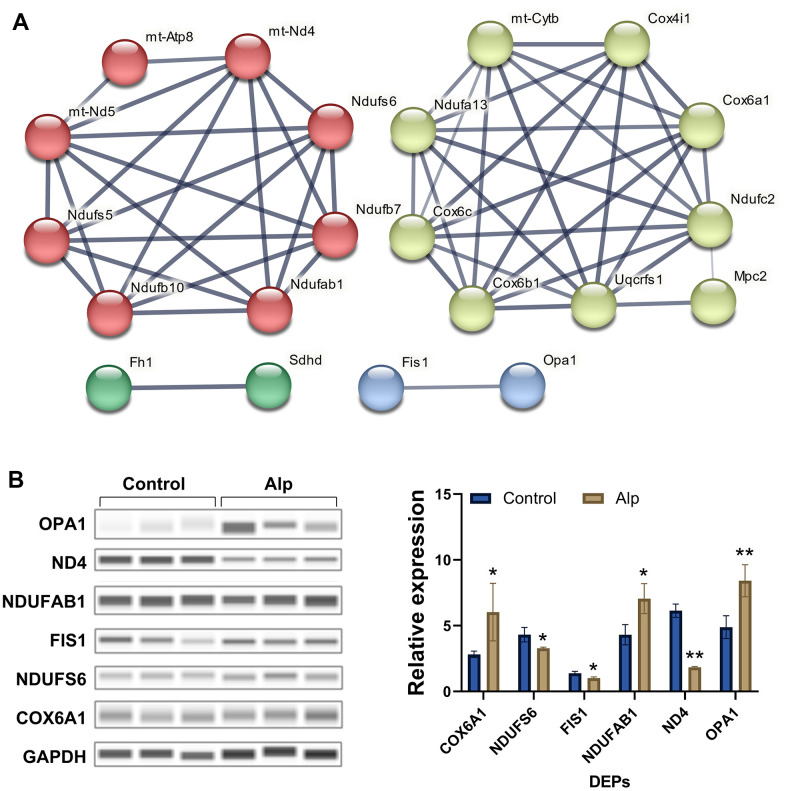
**Network analysis visually and validation of mitochondria-associated proteins.** (**A**) Mitochondria-associated proteins interaction network. The thickness of the line indicates the confidence of interaction. Different marker colors indicate varied clusters of protein function (Red represents Complex I, green represents Complex II, yellow represents Complex IV, and blue represents dynamics process); (**B**) Validation of selected mitochondria-associated proteins, n = 3, Data are presented as mean ± SEM, ^*^p < 0.05, ^**^p < 0.01 vs. control.

The protein network analysis of significantly differentiated proteins (Figure 2A) revealed that these are mitochondrial complex I-related proteins [28–30], mitochondrial complex II-related proteins [31], mitochondrial complex IV-related proteins [32, 33], and mitochondrial dynamics-related proteins [34], which interfere with energy metabolism processes primarily by influencing the mitochondrial electron transport chain (mETC) [35]. Most importantly, the 6 DEPS in above proteomic analysis were selected for further Western blotting validation, and the data (Figure 2B) showed that COX6A1 (*p* < 0.05), Ndufab1 (*p* < 0.05) and OPA1 (*p* < 0.01) in the Alp group were significantly up-regulated compared to the control group, conversely, Ndufs6 (*p* < 0.05), FIS1 (*p* < 0.05) and ND4 (*p* < 0.01) were significantly down-regulated. In summary, repeated administration of Alp leads to a disruption of mitochondrial functional proteins in the mouse hippocampus.

### Repeated administration of Alp causes differential expression of related proteins including cycle, mitophagy and cognition

**Figure 3:**
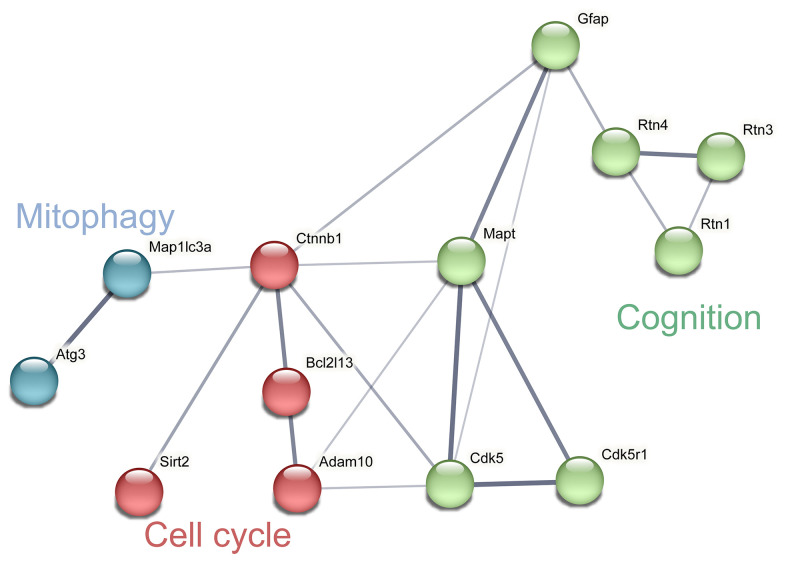
**Network analysis and visualization of related proteins including cycle, mitophagy and cognition.** The thickness of the line indicates the confidence of interaction. Different marker colors indicate varied clusters of protein function (Blue represents mitophagy, red represents cell cycle and green represents cognition).

According to the results of KEGG pathway enrichment (Table 2), key proteins in the apoptosis and cell cycle [36–38], mitophagy [39, 40], and cognitive dysfunction-related [41, 42] pathways were identified (Figure 3). Among these DEPs, the levels of B cell lymphoma 2 (BCL2), catenin beta-1 (CTNNB1), microtubule-associated protein tau (MAPT), reticulon 4 (RTN4), reticulon 3 (RTN3), reticulon 1 (RTN1), and a disintegrin and metalloproteinase domain-containing protein 10 (ADAM 10) were significantly downregulated, while the levels of autophagy-related protein 3 (ATG3), NAD-dependent protein deacetylase sirtuin-2 (SIRT2), microtubule-associated protein 1 light chain 3a (Map1lc3a), cyclin-dependent kinase 5 activator 1 (CDK5R1), cyclin-dependent kinase 5 (CDK5), and glial fibrillary acidic protein (GFAP), were significantly upregulated.

**Table 2:** KEGG terms enriched from significantly regulated cognition-associated proteins between the Alp and control groups.

**Accession No**	**Protein**	**Mw (kDa)**	**FC**	**p-value**
P59017	BCL2	46.7	0.784	0.00497
Q9CPX6	ATG3	35.8	1.584	0.00048
Q8VDQ8	SIRT2	43.2	1.230	0.00007
Q91VR7	LC3	14.3	1.239	0.00154
P61809	CDK5R1	34	1.883	0.04048
P49615	CDK5	33.3	1.215	0.00380
Q02248	CTNNB1	85.4	0.279	0.00643
P03995	GFAP	49.9	1.321	0.00634
P10637	MAPT	78	0.702	0.02365
Q99P72	RTN4	126.5	0.749	0.01261
Q9ES97	RTN3	25.4	0.759	0.00984
Q8K0T0	RTN1	83.5	0.758	0.01975
O35598	ADAM10	83.9	0.044	0.00016

### Alp promotes HT22 cell apoptosis by influencing the cell cycle

**Figure 4:**
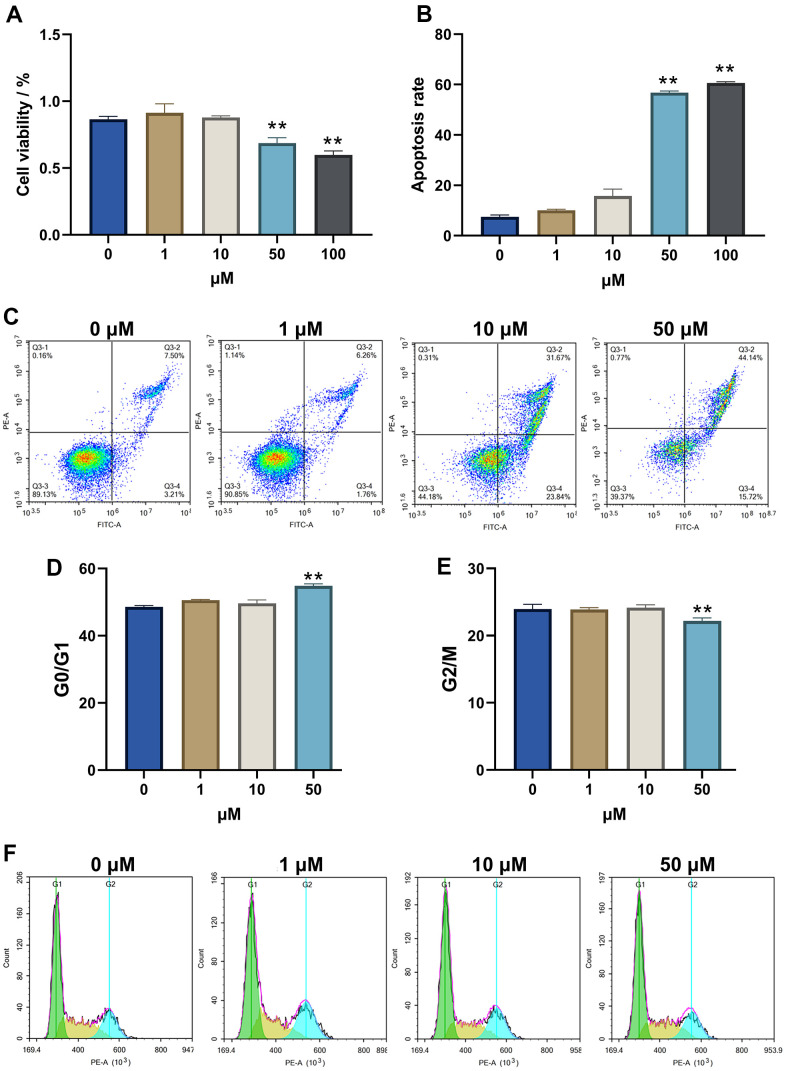
**Alp inhibited the viability and proliferation of HT22 cells.** (**A**) The effect of Alp on HT22 cell viability; (**B**) Apoptosis rate of HT 22 cells in each group is shown as bar graphs; (**C**) Apoptotic histograms from each experimental group. (**D**, **E**) Each cell cycle phase in the experimental group. (**F**) Representative histogram indicating cell cycle arrest; n = 6, Data are presented as mean ± SEM, ^*^p < 0.05, ^**^p < 0.01 vs. control.

Alp showed a decline in HT22 cell viability in a dose-dependent manner. At 48 h, HT22 cells showed 68.74 ± 5.32% and 59.85 ± 5.23% viability for 50 and 100 μM, respectively (Figure 4A). The lowest concentration of Alp (50 μM) that causes cytotoxicity was subsequently used for further experiments.

To understand the neurotoxicity of Alp-induced HT22 cells, the effects of 50 μM Alp on apoptosis and cell cycle analysis were investigated using flow cytometry. The results showed that after treatment with 50 μM Alp, the percentage of apoptosis was significantly higher than in the control group (*p* < 0.01) (Figure 4B, 4C).

In addition, Alp can induce HT22 cellular neurotoxicity by interfering with cell cycle distribution, leading to cell cycle arrest. Compared to the control group, Alp significantly stagnated cells in the G1 phase and reduced cell distribution in the S phase and G2/M phase (*p* < 0.01) (Figure 4D–4F).

The above results suggested that high concentrations of Alp inhibit the proliferation of HT22 cells and promote apoptosis by inducing G1 phase arrest in cells.

### Alp induces mitochondrial dysfunction of HT22 and hippocampal neurons

**Figure 5:**
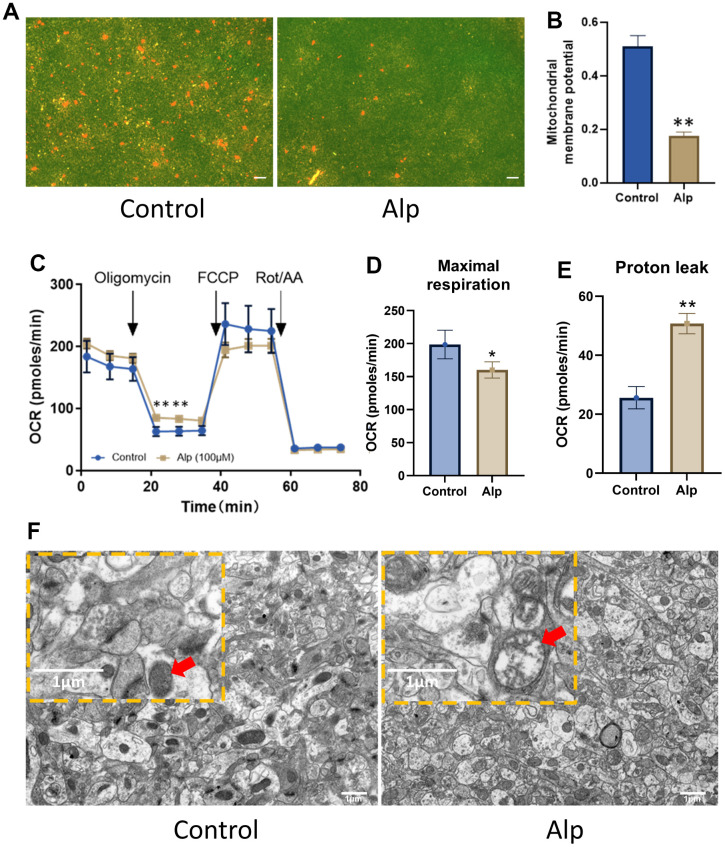
**Effects of Alp on mitochondrial function in the hippocampal neurons.** (**A**, **B**) Alp causes changes in MMP (n = 6). (**C**) Mitochondrial OCR was assessed 48 h post-treatment with Alp (n = 3). Parameters of mitochondrial function, (**D**) maximal respiration, and (**E**) proton leak, were calculated based on the OCR values. (**F**) Repeated Alp administration (1.84 mg/kg) alters the mitochondrial morphology in mouse hippocampal tissue. Data are presented as mean ± SEM, ^*^p < 0.05, ^**^p < 0.01 vs. control. (**A**) Scale bar is 10 μm. (**B**) scale bar is 1 μm.

When the MMP was high, JC-1 polymer in the mitochondrial matrix presented red fluorescence, and at low MMP, JC-1 polymer transformed into monomers, which was green fluorescence. The results of mitochondrial fluorescence probe detection showed that the red fluorescence was evenly distributed in the control group of cells, while the red fluorescence was only little in the cells of the Alp group (Figure 5A), indicating that the MMP of the Alp group was significantly reduced compared to the control group (Figure 5B). A decrease in MMP is a significant event of apoptosis [43]. The results of this experiment suggested that Alp-induced apoptosis of HT22 cells may be related to Alp-mediated reduction of MMP.

To further explore how Alp affects mitochondrial function, its effect on cellular energy metabolism in the primary hippocampal neurons was investigated. The results of mitochondrial stress tests are shown in Figure 5C. The basal OCR was the cellular OCR before the addition of 1.5 μM oligomycin at 15 min. FCCP (2 μM) and the oxidative phosphorylation coupling agent (2 μM) were added at 35 min to detect the maximum cell OCR. The respiratory reserve function was the difference between the maximum cell OCR and the basal cell OCR. Finally, 0.5 μM rotenone and 0.5 μM antimycin A were added at 50 min to detect the non-mitochondrial respiration of cells. OCR data (Table 3) showed that compared to the control group, 50 μM Alp significantly reduced the maximum respiratory capacity (*p* < 0.05) of neurons and increased their proton drain (Figure 5D, 5E) (*p* < 0.01), which resulted in mitochondrial function damage.

**Table 3:** Effect of Alp on mitochondrial energy metabolism in hippocampal neurons (n = 6).

**Group**	**Non-mitochondrial respiration**	**ATP production**	**Basal respiration**	**Maximal respiration**	**Proton leak**
Control	37.64 ± 3.88	100.52 ± 11.46	136.12 ± 15.24	198.62 ± 21.67	25.59 ± 3.78
Alp	34.63 ± 2.11	95.83 ± 4.42	146.54 ± 5.71	160.07 ± 12.32^*^	50.72 ± 3.45^**^

^*^p < 0.05, ^**^p < 0.01 vs. control, Data are presented as mean ± SEM.

Importantly, the ultrastructure of the mouse hippocampal tissue revealed a whole structure, intact outer membrane, clear inner crest, and uniform internal matrix of the mitochondria in the control group. On the other hand, the number of mitochondria was decreased, the shape became irregular, the inner crest dissolution was missing, most mitochondria appeared as vacuoles, and the membrane was dissolved and connected to each other in the Alp group (Figure 5F).

All these *in vitro* and *in vivo* experimental results suggested that Alp induced mitochondrial damage by disrupting mitochondrial ultrastructure and energy metabolism.

### Repeated administration of Alp induces memory impairment *in vivo*

**Figure 6:**
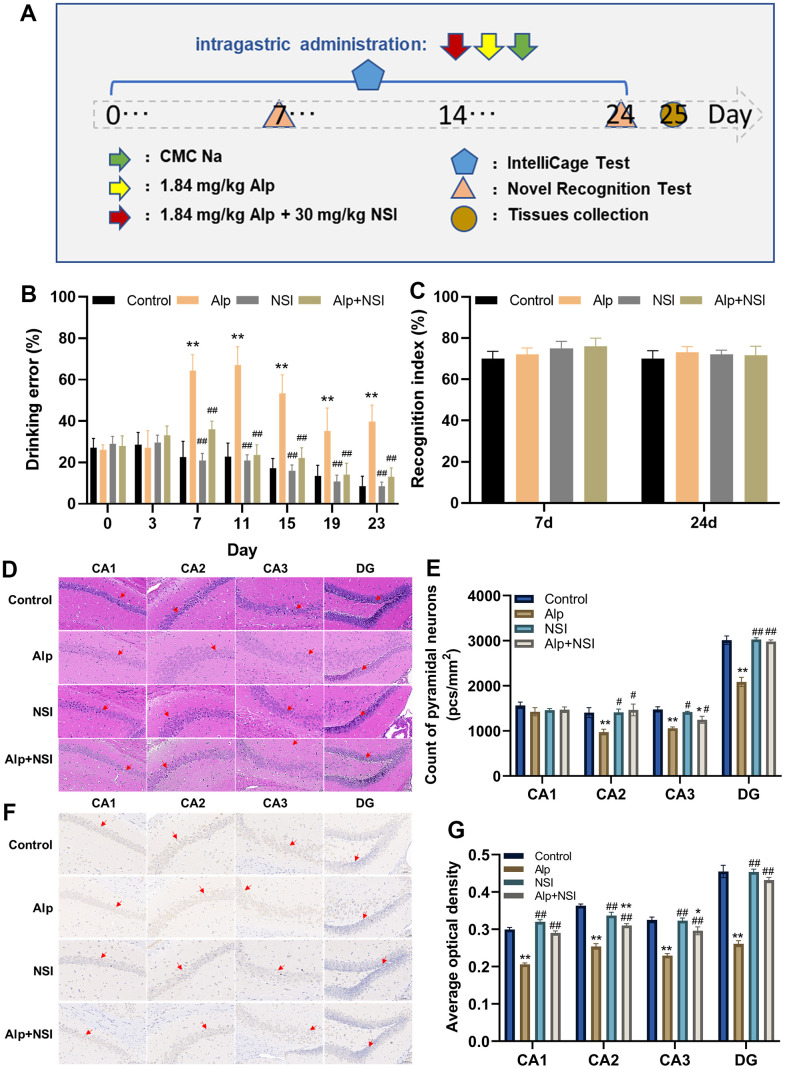
**Effects of repeated administration of Alp on learning and memory in mice.** (**A**) Scheme of behavioral experiments. (**B**) The drinking error of each mouse was examined by ICS (n = 12). (**C**) The learning ability of each mouse was evaluated by NOR (n = 12). (**D**) HE staining in each region (CA1, CA2, CA3 and DG) of the hippocampus. (**E**) Counting of pyramidal neurons in each region (CA1, CA2, CA3 and DG) of the hippocampus (n = 6). (**F**) Immunohistochemical staining in each region (CA1, CA2, CA3 and DG) of the BDNF expression. (**G**) Statistical results of BDNF immunohistochemical in average optical density (n = 6). Scale bar is 100 μm. Data are presented as mean ± SEM. ^*^p < 0.05, ^**^p < 0.01 vs. the control group; ^#^*p* < 0.05, ^##^*p* < 0.01 vs. the Alp group.

The complete behavioral experiment schematic is shown in Figure 6A. The ICS is a fully automated system that assess home cage behavior and cognitive abilities of mice living in a social environment, respectively. This unique experimental setting promotes natural social behavior in a biologically relevant, rich but highly standardized home cage setting. According to our experimental design, all mice learned how to properly drink before the start of the test, although the learning of this protocol was temporary. According to the ICS program, after the experiment begins, each active operation of the mouse, such as visiting a corner, or each passive behavior, such as jet punishment, promotes the mouse to repeatedly learn the program to reduce the attempt to drink water correctly. Therefore, it is easy to explain that mice obtain memory consolidation through continuous reinforcement operations to reduce the drinking error rate. On the contrary, after the memory consolidation process is damaged, the mice will not be able to quickly and effectively reduce the water drinking error rate.

In this ICS test, the drinking error of mice in the Alp group increased significantly from day 5 compared to the control group (*p* < 0.05), although it narrowed gradually (Figure 6B). For the NSI group and the Alp + NSI group, there was no significant difference in the drinking error compared to the control group, but it was significantly reduced from day 7 compared to the Alp group (*p* < 0.01), further indicating that the memory consolidation process in mice was barriered after repeated administration of Alp. Since memory consolidation was impaired, whether repeated administration of Alp affects their learning and acquisition processes needs to be elucidated. Therefore, the learning ability was tested after the re-administration of Alp by NOR test. Interestingly, the results of both the 7^th^ and 24^th^ experiments did not show any significant difference in the NOR index in the Alp group, the NSI group and Alp + NSI group compared to the control group (Figure 6C). Hence, it was speculated that the learning ability of mice is not affected during the 24 days of repeated administration of Alp.

To further demonstrate the effect of Alp administration repeatedly on the process of memory consolidation in mice, pathological changes in the hippocampal neurons were observed, and vertebral neurons were counted. As shown in Figure 6D, 6E, HE staining showed that neurons in the control group were arranged regularly with abundant cytoplasm with round and clear nucleoli. Furthermore, distinct pathological changes, such as neuron necrosis, disordered arrangement, and significantly decreased pyramidal neurons in CA2, CA3, and DG regions were observed in the Alp group (*p* < 0.01). There were no obvious pathological changes in the NSI group, while only minor pathological changes were seen in the CA3 area in the Alp+NSI group. Thus, the mitochondrial protector NSI-189 exerted a protective effect against the above neuronal damage.

Moreover, BDNF is essential for memory consolidation [44], and its expression was evaluated in the hippocampus. The protein expression was significantly downregulated in the hippocampus of mice in the Alp group compared to the control group, further illustrating that repeated administration of Alp impairs the memory consolidation process by reducing the expression of BDNF protein. Moreover, NSI-189 significantly weakens the decline in BDNF expression, thereby protecting the memory consolidation process (Figure 6F, 6G).

Based on these findings, repeated administration of Alp affected the memory consolidation process of learning and memory in mice, while repeated administration of mitochondrial protector NSI along with that of Alp could significantly offset the adverse effects of repeated Alp administration on memory consolidation.

## DISCUSSION

Several clinical studies on Alp have shown that repeated use of Alp could adversely affect cognitive function in patients [45], causing disorders such as memory loss [46, 47], and the extent to which cognitive function recovers after benzodiazepine tapering is unclear. The other clinical trials have shown that low-dose Alp (0.5 mg) mainly alters visual attention function, while high-dose Alp (1.0 mg) can lead to working memory impairment [48]. Therefore, the ED_50_ value of Alp’s hypnotic effect on C57 mice was first determined by a corrected reflex test combined with electroencephalogram and electromyogram analysis; subsequently, this dose was administered continuously. Based on a large number of research reports, we attempted to explore the mechanism of repeated administration of Alp disturbed the process of learning memory.

The proteomics techniques with high-throughput mass spectrometry sequencing features are increasingly used in drug mechanism research [49]. Therefore, proteomic analysis was employed to analyze the DEPs of hippocampus in mice between Alp and control group.

Proteomic results showed that a large number of DEPs were enriched in mitochondrial metabolism pathways, suggesting abnormal mitochondrial energy metabolism. First, significant changes complexes I (Ndufc2 [50, 51], Ndufs5, ND4 [29, 52]), complexes IV (COX 4 [53, 54], COX 6), and ATP 8 [55] indicated mETC disorders. Second, differential expression of mitochondrial fission protein FIS1 [56] and fusion protein OPA1 [57, 58] suggested that mitochondrial homeostasis is disrupted. Similarly, Alp activates the apoptosis pathway by inhibiting the expression of the anti-apoptotic protein BCL2 [59]. Furthermore, the expression differences in autophagy-related proteins, ATG3 [60] and LC3 [61], indicated that mitochondrial autophagy function is impaired. In summary, mitochondrial dysfunction caused by repeated administration of Alp reflected the damage to the mitochondrial respiratory chain, mitochondrial dynamics, and mitochondrial autophagy.

In order to verify the above DEPs and further investigate the effect of Alp on cell and mitochondrial function, HT22 cells were incubated with 50 μM Alp for 48 h, which exerted a significantly harmful effect on the cell cycle and induced apoptosis. Furthermore, Alp significantly reduced the MMP of HT22 cells. Some studies have shown that MMP collapse induces mitochondrial permeability transition (MPT), which in turn releases harmful proteins, such as pro-apoptotic factors, into the cytoplasm, thereby promoting apoptosis or necrosis [62, 63]. In addition, changes in mitochondrial energy metabolism, such as decreased maximal respiration, indicate damage to the mitochondrial respiratory chain and abnormal energy metabolism [64]. Lastly, by the mitochondrial ultrastructure, the appearance of swollen [65] mitochondria and autophagic bodies [66] in the brain tissues of mice in the Alp group reflected the mitochondrial dynamics imbalance and impaired autophagy function.

Several studies have focused on the effects of mitochondrial dysfunction in learning and memory. The proteomic results showed significant changes in the levels of cognition-related proteins, such as rising GFAP [67], falling MAPT [68] and RTN1/3/4 [69]. The increase of GFAP concentration in early plasma in AD patients suggested that it can be used as a marker for the initial diagnosis of AD [70]. MAPT plays an important role in promoting microtubule assembly and stabilizing microtubules, and the accumulation of its hyperphosphorylation induces synaptic toxicity and cognitive impairment [71].

Based on the above results, we speculated that Alp may impairs memory consolidation by causing mitochondrial dysfunction.

Herein, we selected a mitochondrial protector, NSI-189, which significantly increases the total neurite growth and mitochondrial function in normal or diabetic rat sensory neurons [72]. In the mouse models of type 1 and type 2 diabetes, oral administration of NSI-189 (10 mg/kg or 30 mg/kg) prevented small fibrous peripheral neuropathy, promoted hippocampal neurogenesis, synaptic markers, and volume, and protected long-term memory [73]. In a rat model of stroke, oral administration of NSI-189 phosphate (30 mg/kg) significantly improves stroke-induced motor and neurological deficits and stimulates brain remodeling [74]. Thus, a dose of 30 mg/kg of NSI-189 was administrated together with repeated administration of Alp in mice.

Memory acquisition is the first step in memory learning. Memory consolidation is the process by which memories are further stabilized and encoded in neural circuits after they are acquired. In behavioral experiment, ICS and NOR were conducted to evaluate the ability of memory consolidation and acquisition, respectively. Compared with classic cognitive evaluation instruments such as Morris water maze and Y maze, the advantage of ICS is that the behavioral data of mice were effortlessly obtained in non-stress, and the interference of the experimental environment was completely avoided in the complete protocol [75]. By designing an experimental program that automatically changes the position of drinking water, mice need to continuously reinforce temporarily learned drinking memories to avoid the punishment with drinking water incorrectly. Clearly, the drinking error of mice in the control and the Alp+NSI group was decreased with increasing time, which illustrated that their memory of drinking correctly was continuously reinforced, on the contrary, the memory consolidation process of mice in Alp group was significantly blocked. On the other hand, interestingly, the results of NOR tests showed that repeated administration of Alp does not affect the ability of mice to acquisition. In addition, in order to verify the cellular rescue effect of NSI alone on Alp-induced cell dysfunction *in vitro*, the levels of 3 DEPs including ND4, NDUFAB1, NDUFS6 were detected at the HT22 cells by WB (Supplementary Figure 3). *In vitro* results also showed that the mitochondrial protector NSI-189 had a rescue effect on Alp-induced cell dysfunction, consistent with *in vivo* results.

Although this study also has limitations, only assessing hippocampus-dependent spatial memory and excluding other types of memory such as fear memory, it still provides a unique mitochondrial protection strategy for intervening in memory consolidation with repeated administration of Alp.

## CONCLUSIONS

**Figure 7:**
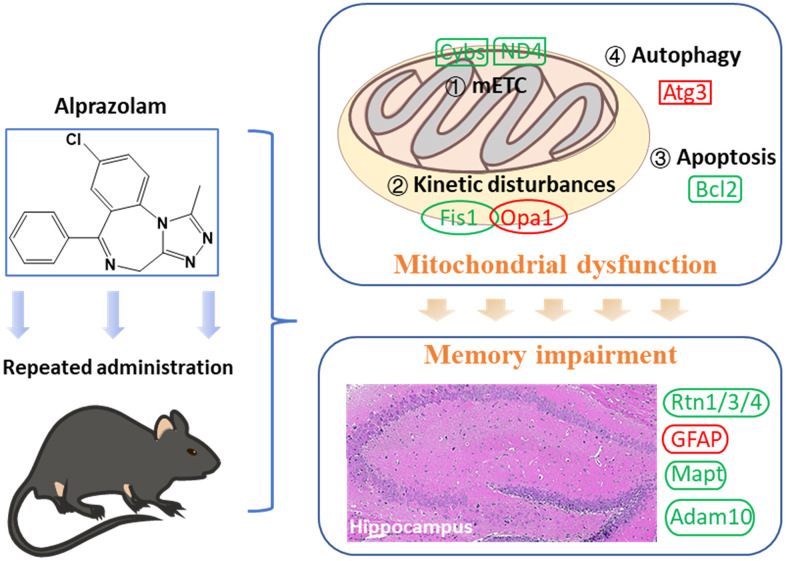
Mitochondrial dysfunction following repeated administration of alprazolam causes attenuation of hippocampus-dependent memory consolidation in mice.

Repeated administration of Alp causes attenuation of hippocampus-dependent memory consolidation rather than memory acquisition, and this adverse effect mediated by mitochondrial dysfunction. Nevertheless, the neurotoxicity can be prevented by mitochondrial protectors, which provides new ideas for the clinical administration of Alp (Figure 7).

## MATERIALS AND METHODS

### Cell culture and treatment

HT22 cell line (Procell, Wuhan, China) was cultured in DMEM (Gibco, USA) supplemented with 10% fetal bovine serum (FBS) and 1% penicillin/streptomycin (Life Technologies, USA). All cell cultures were incubated at 37° C under 5% CO_2_ in a humidified atmosphere.

### Animals and treatments

Adult C57BL/6J male mice (6-week-old, 18 ± 1 g) and neonatal C57BL/6J mice (within 24 h) were obtained from HFK Biotechnology Co., Ltd (Beijing, China).

Before any behavioral and experimental procedures, mice were housed (3 animals per cage) with food and water available ad libitum at 24° C on a 12 h light/dark cycle (lights on at 7 a.m.). The animal welfare and experimentation program were reviewed and approved by the Animal Ethics Committee of State Key Laboratory of NBC Protection for Civilian (LAE-2022-03-001).

In behavioral tests, mice (4-week-old) were divided into the following groups with different treatments (12 mice per group): (1) the control group with 1% sodium carboxymethylcellulose CMC Na (Sigma-Aldrich, USA); (2) the Alp group (Alp) treated with 1.84 mg/kg (ED_50_, Supplementary Figure 1) Alp (Yimin Pharmaceutical Co., Ltd, China) suspended with 1% CMC Na; (3) the NSI group (NSI) treated with 30 mg/kg NSI-189 (Selleck, USA) suspended with 1 % CMC Na; (4) the Alp and NSI-189 group (Alp + NSI) treated with 1.84 mg/kg Alp and 30 mg/kg NSI-189 suspended in 1% CMC Na. All mice were intragastrically administered once a day from 9:00 a.m. to 10:00 a.m. for 24 consecutive days. After the last administration, all mice were anesthetized with isoflurane and rapidly decapitated, and all tissue were collected.

### Proteomics analysis

In proteomic analysis experiments, mice were (6-week-old) divided into two groups (6 mice per group, and all mice were treated by intragastric administration): (1) the control group with 1% CMC Na; (2) the Alp group (Alp) treated with 1.84 mg/kg Alp suspended with 1% CMC Na. All mice were continuously administered for 24 days. After the last administration, all mice were anesthetized with isoflurane and rapidly decapitated on ice, and the fresh hippocampus tissues were collected immediately and stored at −80° C.

Subsequently, above fresh hippocampus samples were placed on ice. The protein lysis buffer containing 1% protease inhibitors and 1% phosphatase inhibitors was added to the tissues and homogenized three times for 1 min each with a tissue grinder. The protein concentration in the sample supernatant was determined by the BCA protein assay kit (Thermo Fisher Scientific, USA).

An equivalent of 100 μg protein was resuspended in 100 mM triethylammonium bicarbonate buffer (TEAB). Trypsin was added to the protein at a trypsin-to-protein mass ratio of 1:50 and incubated at 37° C overnight. An equal amount of trypsin-digested peptide was taken from each sample and resuspended in UPLC loading buffer after vacuum drying (Phase A: 2% acetonitrile, pH 10; Phase B: 80% acetonitrile, pH 10). The mixed peptides were fractionated by Vanquish Flex binary UHPLC chromatography (Thermo Fisher Scientific, USA) on a Acquity UPLC BEH C_18_ Column (1.7 μm, 2.1 mm × 150 mm, Waters, USA) to increase the proteomic depth. The peptides were redissolved in a loading buffer (2% ACN with 0.1% formic acid) and analyzed by online nanoflow liquid chromatography-tandem mass spectrometry on an EASY-nLC system (Thermo Fisher Scientific, USA) connected to a Q Exactive HF-X quadrupole orbitrap mass spectrometer (Thermo Fisher Scientific, USA).

The Q Exactive HF-X instrument was operated in the data-independent acquisition mode (DIA) to automatically switch between full scan MS and MS/MS acquisition. The survey of full scan MS spectra (m/z 300–1500) was acquired; then, the all-precursor ions were selected into collision cell for fragmentation by high-energy collision dissociation (HCD). DIA was performed with a variable isolation window; each window overlapped by 1 m/z for a total of 40 windows. The DIA data files were analyzed using Spectronaut (Biognosys AG, Version 14, Switzerland) with the default settings, and the retention time prediction type was set to dynamic iRT.

The proteomics statistical analysis was performed as described previously [19]. Proteomic data was analyzed using the one-way analysis of variance (ANOVA), followed Tukey’s post-hoc test. Statistically significant parameters were selected with fold-change (FC) > 1.2 (or < 0.83) and *p* < 0.05 for the proteomic analysis. DEPs were used for Gene Ontology (GO) and Kyoto Encyclopedia of Genes and Genomes (KEGG) enrichment analysis. Protein-protein interaction analysis was carried out using String v13.5.

### Cell viability

HT22 cells were seeded in the 96-wells at a density of 1 × 10^4^ cells/well and incubated overnight at 37° C with 5% CO_2_. Then, they were treated with various concentrations (0, 1, 10, 50, and 100 μM, dissolved with 0.5% DMSO, 100 μL) of Alp for 48 h. The Cell Counting Kit-8 (CCK-8, Solarbio Science and Technology Co., Ltd, Beijing, China) reagent was added to each well and incubated at 37° C for 1 h with 5% CO_2_, and the absorbance of the samples was measured in a microplate reader (Spark, Tecan, Switzerland) at 450 nm.

### Flow cytometry

HT22 cells were plated in 6-well plates at a density of 1.5 × 10^6^ cells per well and treated with drugs for 48 h. Then, cells were trypsinzed and harvested by centrifugation, followed by staining with Annexin V-FITC/PI kit (556547, BD Biosciences, USA); the cells were incubated with Annexin V-FITC and PI at room temperature for 15 min in the dark. The apoptosis rate was examined by flow cytometry (NovoCyte, Agilent, USA) in 30 min. The experiment was repeated three times with four duplicate wells for each group.

Each group of well-grown cells was washed twice with phosphate-buffered saline (PBS), resuspended with pre-chilled in 70% ethanol, fixed at 4° C for 12 h, permeabilized with 0.1% Triton X-100, stained with PI/RNase (550825, BD Biosciences, USA) in PBS, and incubated at 37° C in the dark for 30 min. Subsequently, the cell cycle distribution of each group was detected 3 times by flow cytometry, and the experiment was repeated 3 times.

### Mitochondrial activity assay

The JC-1 fluorescent probe (Beyotime, China) was used to detect mitochondrial membrane potential (MMP). HT22 cells were plated in 6-well plates at a density of 1.5 × 10^6^ cells per well and treated with drugs for 48 h. After treatment, the culture medium was discarded, the cells were washed with PBS once, and 1 mL of medium and 1 mL of JC-1 staining solution was added to each well, and incubated for 20 min. Subsequently, the supernatant was discarded, the cells were washed two times with JC-1 staining buffer, and then 2 mL of medium was added. Finally, the images from each well were acquired using a fully automated cell imaging multifunctional microplate detector (Cytation 1, Bio-Tek, USA).

### Hippocampal primary neurons culture *in vitro*

The extraction and *in vitro* culture of primary cells are based on previous studies [76]. The hippocampal tissue was digested by papaya enzyme (P123425, Aladdin, USA) and filtered through a 40-μm cell sieve to prepare a single-cell suspension. The neurobasal-A culture medium (2429791, Gibco, USA) containing 2% B27 supplements (2415388, Gibco, USA) and 25 μM glutamine (3505061, Gibco, USA) was added to single-cell suspension. The cell culture medium was changed every 3 days, the old medium was aspirated and the neurons were cleaned 3 times by PBS, and finally the fresh medium was added. After 7 days of culture in a humidified atmosphere of 5% CO_2_/95% air at 37° C, cell experiments were performed subsequently.

### Mitochondrial bioenergetics analysis

Primary hippocampal neurons were seeded in 96-well plates at a density of 1.2 × 10^4^. After 24 h of culture with 5% CO_2_ at 37° C, the neurons were randomly divided into two groups, control and Alp, 6 wells each. The neurons in the Alp group were treated with 100 μL medium containing the 50 μM Alp, while the control group were treated with normal medium. After 48 h, neurons were washed with assay solution (culture medium containing 1 mM sodium pyruvate, 2 mM glutamine, and 10 mM glucose) and cultured at 37° C for 45 min without CO_2_. The oxygen consumption rate (OCR) was measured by cell energy metabolism analyzer (Seahorse XFe, Agilent Technologies, USA). 20 μL Oligomycin (1.5 μM), 20 μL carbonyl cyanide 4-(trifluoromethoxy) phenylhydrazone (FCCP, 2 μM), and 25 μL rotenone/antimycin A (R/A, 0.5 μM) were dissolved with assay solution and injected sequentially during the assay. Each sample was tested three times. Finally, basal breathing, ATP-related breathing, and maximal and spare respiratory capacity were determined, respectively.

### Automated Western blotting

Proteins levels in issues or cells were measured using the Jess western blotting system (ProteinSimple, USA), which is an automated capillary-based size separation and nano-immunoassay system. Jess-specific-reagents (PS-ST05EZ, ProteinSimple), including their rabbit secondary antibodies, were used according to the manufacturer’s protocols. The rabbit anti-GAPDH (ab181602, Abcam), anti-COX6A1 (11460-1-AP, Proteintech), anti-NDUFS6 (ab230464, Abcam), anti-FIS1 (BPS04232S, Abmart), anti-NDUFAB1 (BT55373S, Abmart), anti-ND4 (26736-1-AP, Proteintech) and anti-OPA1 (BPS06076S, Abmart) were diluted at 1:50 in antibody diluent. Digital image of chemiluminescence of the capillary was captured with Compass Simple Western software (version 6.2.0, ProteinSimple) that calculated automatically area (chemiluminescence intensity).

### Intelligent cage system (ICS) test

The cognitive function of mice was evaluated by the ICS (New Behavior, TSE Systems, Germany) as described previously. The experimental protocol was further improved by optimizing the program design. The whole 43-day protocol includes intelligent programming, mouse drinking training, and mouse behavior testing, and the details of the experiment are provided in the Supplementary Figure 2.

### Novel objects recognition (NOR)

Before starting the tests, each mouse was allowed to explore freely in an open field for 10 min. After 30 min, the mice were placed again in the field to explore freely for 10 min, at which point two identical wooden cylinders were placed symmetrically in the field. After 1 h, one of the wooden cylinders was replaced with a plastic cone with an equal base area (defined as a new object), and the mouse was re-entered into the field to explore freely for 10 min. The time spent exploring each object during the training and testing periods was recorded (VisuTrack, XinRuan, China) separately, and the novel object recognition index was calculated. The novel object recognition index is the percentage of time spent exploring a new object with respect to the time spent exploring two objects.

### Hematoxylin-eosin (HE) staining

Hippocampus slices in the ischemic brains were used to observe the changes in neuronal cell morphology by HE staining. All information on tissue sections was scanned and imaged using the slide scanner (Pannoramic, 3DHISTECH, Hungary). The scanning software CaseViewer2.4 (3D-HISTECH, Hungary) was applied to select the tissue target area for 400× imaging, followed by Image ProPlus 6.0 (version 6.0, Media Cybernetics, USA) to count the number of pyramidal neurons in each image, and the number of pyramidal neurons was calculated per unit area.

### Immunohistochemistry

The paraffin slices of the hippocampal brain were blocked with 3% bovine serum albumin (BSA) at 37° C for 30 min and then incubated overnight with 1% brain-derived neurotrophic factor (BDNF) antibody (ab108319, Abcam) at 4° C. The standard protocols were followed as described previously [77]. The different regions of the hippocampus were selected to acquire images under a 400× field of view (Eclipse E100, Nikon, Japan). Three non-overlapping regions were randomly selected for each slice, and the Image ProPlus 6.0 image analysis system was used to determine the average optical density values of BDNF-positive cells in each region of the hippocampus under different fields of view.

### Transmission electron microscopy (TEM)

The hippocampus tissue was fixed in 1% osmium acid prepared in 0.1 M phosphoric acid buffer PB (pH 7.4) at room temperature for 2 h. Then, the tissue was stained with 2% uranyl acetate in the dark, dehydrated in an acetone gradient solution, and embedded in an epoxy resin. The resin block was used to prepare 60-nm ultrathin sections on an ultramicrotome (UC7, Leica, Germany) and stained with 2.6% lead citrate and 2% uranium acetate. Finally, images of mitochondrial ultrastructure were acquired by transmission electron microscopy (HT7800, Hitachi, Japan).

### Statistical analysis

GraphPad Prism 9.0 (GraphPad Software, USA) was used for data analysis. The data conformed to a normal distribution, which was expressed as the mean ± standard error of the mean (SEM). An unpaired t-test was used to compare two groups, and one-way or two-way ANOVA was used to compare multiple groups. *p* < 0.05 was considered statistically significant difference.

### Data availability statement

The detailed data used to support the findings of this study are available from the corresponding author upon written request.

## Supplementary Material

Supplementary Materials and Methods

Supplementary Figures

Supplementary Table 1
