# Supplementary material for: Mitochondrial dysfunction following repeated administration of alprazolam causes attenuation of hippocampus-dependent memory consolidation in mice
Source: Aging (Albany NY). 2023 Oct 5;15(19):10428–52. doi: 10.18632/aging.205087 (PMC10599724; doi:10.18632/aging.205087)
Supplement: Supplementary Materials and Methods [file aging-15-205087-s001.pdf]

SUPPLEMENTARY MATERIALS AND METHODS

ED50 value of Alp causing hypnotic effects in C57 mice

C57 mice were randomly divided into 6 groups (8 per group), each mouse in a different dose group was administrated a different dose (1,1.2,1.6,2.0,2.2,2.4 mg/kg) of Alp suspension. To assess the hypnotic effect of Alp, the time of loss of the righting reflex (LORR, as a measure of unconsciousness) was recorded in hypnotic experiments [1, 2]. In this experiment, if the LORR time of mice after administration can exceed 10 min, this phenomenon was defined as falling asleep, and the sleep rate of mice induced by different doses of Alp was calculated (Supplementary Figure 1A). Moreover, Alp induced asleep in a dose-dependent fashion with a 50% effective dose of 1.84 mg/kg (Supplementary Figure 1B).

C57 mice were randomly divided into 2 groups (6 per group), the two groups of mice were embedded with electrodes to monitor EEG and EMG within 1 h after administration of 1% CMC Na or 1.84 mg/kg Alp. Referring to the information processing method [3], through the comprehensive analysis of EEG and EMG, the sleep-wake cycle was divided into waking, slow wave sleep (SWS), and rapid eye movement sleep (REMS). Sleep phase analysis data (Supplementary Table 1) showed that mice in the Alp group showed longer sleep time after 1 h of administration compared to the control group.

Intelligent cage system (ICS) test

In this study, the IntelliCage cage (33 cm × 55 cm × 20 cm) consisted of four symmetrically distributed corners of the same structure and a central resting area (blue circular area in Supplementary Figure 2A) where food is served, and each corner comprising a gate (yellow fan-shaped area in Supplementary Figure 2A) and two drinking windows (black ring area in Supplementary Figure 2A), where the dotted line indicates that the gate or drinking window is open and the solid line indicates that it is closed.

During the acclimatization period of 4 days, the animals were housed in IntelliCage system containing standard sawdust bedding. The mice were allowed free access to food and water throughout the acclimatization period and were housed under light-dark conditions (lights on: 08:00–20:00), an ambient temperature of 22 ± 10° C, and 50–60% relative humidity.

After 4 days of the initial acclimatization period, the transponders were injected into the scruff on the neck of every mouse anesthetized by isoflurane. Importantly, the mouse signalers were tested sequentially to ensure that their subtle behavior, such as visiting and nose-poking, could be detected quickly and sensitively by the detector. Next, the door was opened and the mice were allowed to freely explore the environment and drink water for 3 days. Then, all doors were closed and mice were required to complete the nose-poke to open the door to drink water for 3 days. The number of corner visits and nose-pokes was monitored to assess mouse corner preferences. Then, in the position allocation phase, the least preferred corner (corner 1 in Supplementary Figure 2B) of the mouse was designated as "correct," and conversely, the preferred corner (corners 1, 2, and 3 in Supplementary Figure 2B) was "wrong." Although the mice were able to visit any corners, only when the corner was "correct," the door could be opened and the mice allowed to drink. After the mouse entered the "correct corner, the green LED light (green area in Supplementary Figure 2B) flared up, and the mouse opened the drinking window by three consecutive nose-pokes, allowing the animal to drink water freely for 10 s. Conversely, if the mouse entered the "wrong" corner, the red LED light flared up (red areas in Supplementary Figure 2B). When the mouse nose-poked any drinking windows, the gas valve was triggered to quickly eject gas (white explosive shapes in Supplementary Figure 2B to punish the mouse). Interestingly, if the mouse entered the corner correctly (corner 1 in Supplementary Figure 2B), the next "correct" corner would be automatically replaced by the next corner rotated clockwise (corner 2 in Supplementary Figure 2C). If the mouse entered the corner wrongly (corners 2, 3, and 4 in Supplementary Figure 2B), all corners' positions are retained (Supplementary Figure 2B).

After 3 days of professional training, the learning ability of each mouse was evaluated, and finally, 36 mice were randomly divided into 3 groups, with 12 animals in each group for drug treatment. The dosing cycle lasted 24 days, and the daily activity parameters of each mouse, such as the error rate of drinking, were recorded sequentially. The complete experimental protocol described above is shown in Supplementary Figure 2D.

NSI rescue effect on Alp-induced cell dysfunction

HT22 cells were seeded in the 6-wells at a density of 1.5×10^5 cells/well. After 24 h of culture with 5% CO2 at 37° C, the cells were randomly divided into 4 groups with Control, Alp (50 μM Alp), NSI (5 μM NSI) and

Alp+NSI (50 μM Alp and 5 μM NSI) group, 6 wells per group with a 100 μL medium per well. After 48 h, the total protein of above groups of cell samples was extracted for the next Western blotting assay.

WB results showed that compared with the control group, NDUFS6 and ND4 in Alp group were significantly down-regulated, and NDUFAB was significantly up-regulated. The results were significantly opposite in the alone NSI group, moreover, NSI significantly improved the protein imbalance caused by 50μM Alp.

SUPPLEMENTARY REFERENCES

1. Katayama S, Irifune M, Kikuchi N, Takarada T, Shimizu Y, Endo C, Takata T, Dohi T, Sato T, Kawahara M. Increased gamma-aminobutyric acid levels in mouse brain induce loss of righting reflex, but not immobility, in response to noxious stimulation. Anesth Analg. 2007; 104:1422–9, table of contents. https://doi.org/10.1213/01.ane.0000261519.04083.3e PMID:17513635

2. Li JY, Gao SJ, Li RR, Wang W, Sun J, Zhang LQ, Wu JY, Liu DQ, Zhang P, Tian B, Mei W. A Neural Circuit from the Paraventricular Thalamus to the Bed Nucleus of the Stria Terminalis for the Regulation of States of Consciousness during Sevoflurane Anesthesia in Mice. Anesthesiology. 2022; 136:709–31. https://doi.org/10.1097/ALN.0000000000004195 PMID:35263424

3. McShane BB, Galante RJ, Biber M, Jensen ST, Wyner AJ, Pack AI. Assessing REM sleep in mice using video data. Sleep. 2012; 35:433–42. https://doi.org/10.5665/sleep.1712 PMID:22379250
