# Supplementary material for: Mitochondrial dysfunction following repeated administration of alprazolam causes attenuation of hippocampus-dependent memory consolidation in mice
Source: Aging (Albany NY). 2023 Oct 5;15(19):10428–52. doi: 10.18632/aging.205087 (PMC10599724; doi:10.18632/aging.205087)
Supplement: Supplementary Figures [file aging-15-205087-s002.pdf]

SUPPLEMENTARY FIGURES

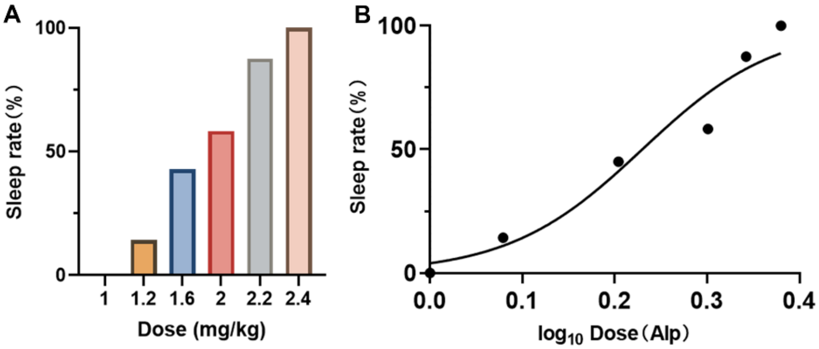

Supplementary Figure 1. The dose-sleep rate change (**A**) and ED50 curve (**B**) of hypnotic effect induced by different doses of Alp in mice. (n=8).

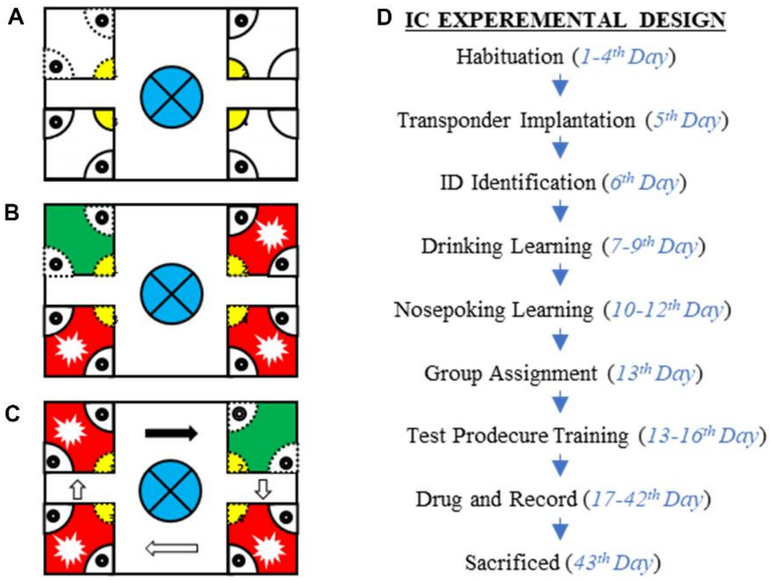

Supplementary Figure 2. Structure and experimental program design of IntelliCage (**A**–**C**) and Intelligent cage system test protocol (**D**).

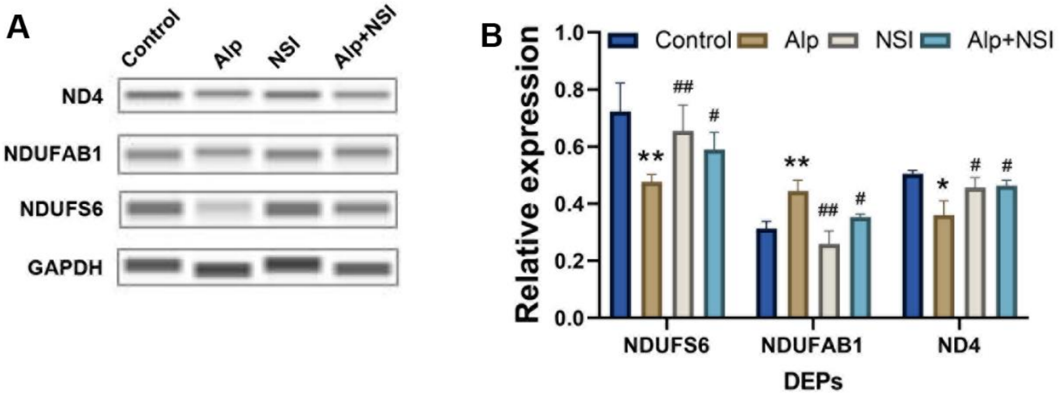

Supplementary Figure 3. Western blotting detected the results of ND4, NDUFAB1, NDUFS6 in each group. (**A**) Protein banding results plot for ND4, NDUFAB1, NDUFS6 in each group; (**B**) Statistics of relative protein expression of ND4, NDUFAB1 and NDUFS6 in each group. n = 3, Data are presented as mean ± SEM, $^{*}p < 0.05$, $^{**}p < 0.01$ vs. control, $^{\#}p < 0.05$, $^{\#\#}p < 0.01$ vs. the Alp group.
