# Supplementary material for: Mitochondrial dysfunction following repeated administration of alprazolam causes attenuation of hippocampus-dependent memory consolidation in mice
Source: Aging (Albany NY). 2023 Oct 5;15(19):10428–52. doi: 10.18632/aging.205087 (PMC10599724; doi:10.18632/aging.205087)
Supplement: Supplementary Table 1 [file aging-15-205087-s003.pdf]

SUPPLEMENTARY TABLE

Supplementary Table 1. Sleep phase of two group of mice (n=6).

Group	Waking (%)	SWS (%)	REMS (%)
Control	93.3±1.5	5.2±1.1	1.3±0.9
Alp	78.8±3.7*	15.7±3.3*	4.2±1.5*

$^{*}p < 0.05$ vs. control, Data are presented as mean ± SEM.
